# Alpha coma evolving into spindle coma in a case of acute fulminant hepatic failure: What does it signify?

**DOI:** 10.4103/0972-2327.61285

**Published:** 2010

**Authors:** Abhijit Das, Ajith Cherian, G. K Dash, Ashalatha Radhakrishnan

**Affiliations:** Department of Neurology, Sree Chitra Tirunal Institute of Medical Sciences and Technology, Trivandrum, India

**Keywords:** Alpha coma, electroencephalogram, hepatic failure, metabolic encephalopathy, spindle coma

## Abstract

A 44-year-old male developed acute fulminant hepatic failure of unknown etiology and expired within four days. His serial electroencephalograms (EEGs) showed diffuse background slowing on day one, which evolved into “alpha coma” and later into “spindle coma” over the ensuing two days. Such EEG transition is hitherto undescribed in patients with hepatic encephalopathy and gives fresh insight into the etiopathogenesis of specific EEG patterns in diffuse encephalopathy.

## Introduction

Electroencephalogram (EEG) changes in hepatic encephalopathy vary from low-frequency alpha rhythm (8 Hz) admixed with bilateral theta activity that may later evolve into theta-delta slowing over both hemispheres, with or without triphasic waves. Presence of alpha coma and spindle coma pattern in the same patient has hitherto not been highlighted in cases of coma due to fulminant hepatic failure. We report this unusual EEG evolution occurring within a span of three days and discuss its possible electrophysiological basis and prognostic implications.

## Case Report

A 44-year-old male with headache and malaise of one-day duration was found unconscious at home. He was last seen by his relative 4-5 hours prior to the present state, ambulant, and verbalizing. He had a history of complex partial seizures of extra temporal origin of 20 years duration with infrequent secondary generalization and was on Phenytoin 350 mg/day. His neuroimaging (MRI brain-1.5T) was normal. On admission, he was febrile with a Glasgow Coma Score of 3/15 without other focal abnormalities on neurological examination and was put on mechanical ventilation. His liver function tests revealed elevated serum glutamic-oxaloacetic transaminase [SGOT =658 U/L, N = <40 U/L] and glutamic-pyruvic transaminase [SGPT = 240 U/L, N = 30-65 U/L]. During serial monitoring they further rose to 50-100 times respectively in the next 48 hours. His total bilirubin was 4.5 mg% [N = 0.2-1.0 mg], with a direct fraction of 3.2 mg% [N = 0.4-0.6 mg%], serum ammonia 65 µmol/L [N = 11-35 µmol/L], alkaline phosphatase 156U/L [N = 50-136 U/L], and albumin 4.1 gm% [N = 4-5 g]. He had progressively worsening coagulation parameters [INR 2.1 to 3.4]. CSF study was normal and blood culture, urine toxic screen (for paracetamol, benzodiazepines, phenothiazines, and phenytoin) and viral markers (for hepatitis A, B, C, E viruses, and HIV I, II) were negative. Ultrasound scan of abdomen and CT scan of brain were normal.

**Figure 1:**
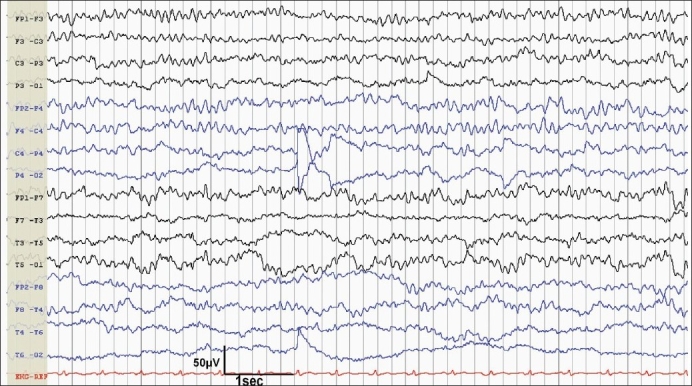
EEG taken on day 2 shows frontally dominant monotonous alpha frequency activity, which is continuous and nonreactive suggesting alpha coma

**Figure 2:**
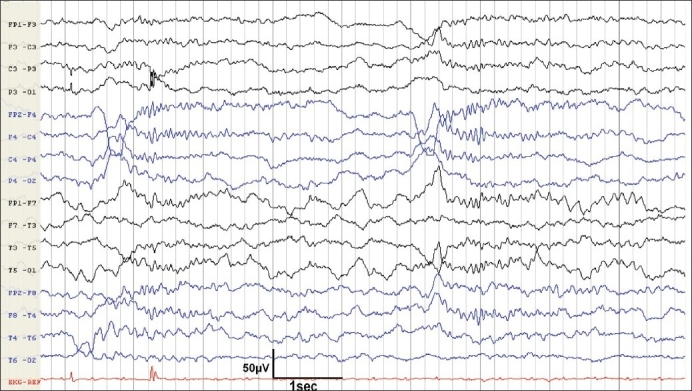
EEG taken on day 3 shows sleep spindles in the range of 12-14 Hz with vertex sharp waves, and K complexes, superimposed on a background of delta and theta activity, in the unarousable patient suggesting spindle coma

Serial EEGs were acquired on daily basis, ranging from forty- five minutes to four hours, but no continuous monitoring was done. On day one, it showed diffuse back ground slowing, which evolved into frontally dominant alpha frequency waves without reactivity, suggestive of “alpha coma” on day 2 [Figure 1]. On the third day of admission, his EEG showed sleep spindles, vertex sharp waves, and K-complexes, occurring periodically throughout the record, without any arousal or reactivity to any external or painful stimuli. This was suggestive of “spindle coma” [Figure 2]. He developed severe coagulopathy and succumbed on the fourth day due to acute fulminant hepatic failure. A postmortem liver biopsy showed degenerative changes with hemorrhagic necrosis and brownish pigmentation of hepatocytes in zone three (perivenular). Periportal hepatocytes were viable and portal area showed scanty inflammatory cells. These findings were nonspecific and did not point towards any exact etiology. Hence, a diagnosis of “fulminant hepatic failure of indeterminate etiology” was made.

## Discussion

EEG changes in hepatic encephalopathy depend on the stage of coma. From low-frequency alpha rhythm (8 Hz) admixed with bilateral theta slowing (early stages), it may later evolve into theta-delta slowing over both hemispheres with or without triphasic waves. With increasing stupor sleep activity disintegrates. In severe coma, arrhythmic delta activity decreases, both in frequency and amplitude, evolving into electrocerebral silence.[1]

Our patient's EEG done on day 1 showed nonreactive, generalized delta slowing, suggestive of a diffuse electrophysiological disturbance. This evolved into “alpha coma pattern” on second day. On the third day, his EEG revealed “spindle coma pattern”. Alpha coma is a term used when a predominant alpha frequency activity is noted in a comatose patient which is not posterior dominant, continuous and nonreactive.[2] Three main groups of patients have been observed to manifest alpha coma: those with diffuse brain insults usually after cardiopulmonary arrest, those with localized brainstem lesions at or just caudal to the pontomesencephalic junction, and those with various toxic or metabolic problems.[3] The exact brain generator of alpha frequency in coma is not clear. Many authors believe that alpha-frequency coma represents a *de novo* abnormal pattern. Animal studies have shown that after hypoxic brain insult, alpha-like activity appears first with maximum amplitude in the amygdala. This activity could be blocked by bilateral ablation of the amygdala, but not by destruction of cerebral hemispheres.[4,5] However, in view of the variety of associations found in alpha coma, some authors are of the opinion that it should be considered a descriptive, not diagnostic, term.[3] In the absence of localizing neurological signs and structural abnormality in brain imaging, the origin of alpha coma in our patient may be contributed to diffuse electrophysiological dysfunction.

Spindle coma is an electroclinical entity in which physiologic sleep patterns, such as sleep spindles in the 12 to 14 Hz range, vertex sharp waves, and K complexes, superimposed on a background of delta and theta activity, occur synchronously in patients with altered consciousness. Spindle coma pattern is distinguished from physiologic slow wave sleep by the inability to rouse the patient to a normal level of consciousness.[6] Head injury, anoxic encephalopathy, viral encephalitis, drug intoxication, and metabolic encephalopathy can result in this pattern. Since human spindle generators are located in the thalamus, the disorder in spindle coma is due to altered function in brain regions caudal to the thalamus, causing alteration of consciousness and impairing the modulation of slow-wave sleep potentials.[7] In our patient, there were no other clinical or imaging evidence for involvement of brain stem functions. Hence, the findings in this case might indicate that these EEG patterns are more of descriptive terms rather than of localizing value. However, in the absence of brain postmortem report, we cannot definitely prove the absence of focal pathology in brainstem that might have contributed to the EEG patterns, but the available evidences speak against it.

Cycling slow wave sleep patterns in EEG have been reported rarely during prolonged nocturnal recordings in patients with alpha coma.[8] Although, we did not have continuous recording done in our patient, the recordings were done in day time for four days consecutively and were persistently having sleep spindles in the range of 12-14 Hz, vertex sharp waves, and K complexes only, without any intervening alpha activity. This would argue against the cyclical sleep pattern.

Previous reports denoted better outcomes with spindle coma rather than alpha coma.[9] However, it has been debated in later reports.[6] The toxic or metabolic subgroup in alpha coma patients also had better outcomes.[3] Hence, solely based on EEG patterns it is often difficult to prognosticate patients with deep coma, as in our case. Such an evolutionary pattern of EEG has hitherto not been highlighted in cases of coma due to fulminant hepatic failure.
